# Flashy flagella: flagellin modification is relatively common and highly versatile among the *Enterobacteriaceae*

**DOI:** 10.1186/s12864-016-2735-x

**Published:** 2016-05-20

**Authors:** Pieter De Maayer, Don A. Cowan

**Affiliations:** Centre for Microbial Ecology and Genomics, University of Pretoria, 0002 Pretoria, South Africa; Department of Microbiology, University of Pretoria, 0002 Pretoria, South Africa

**Keywords:** *Enterobacteriaceae*, Flagellum, Flagellin glycosylation, Methylation, *N-*lysine methylase

## Abstract

**Background:**

Post-translational glycosylation of the flagellin protein is relatively common among Gram-negative bacteria, and has been linked to several phenotypes, including flagellar biosynthesis and motility, biofilm formation, host immune evasion and manipulation and virulence. However to date, despite extensive physiological and genetic characterization, it has never been reported for the peritrichously flagellate *Enterobacteriaceae*.

**Results:**

Using comparative genomic approaches we analyzed 2,000 representative genomes of *Enterobacteriaceae*, and show that flagellin glycosylation islands are relatively common and extremely versatile among members of this family. Differences in the G + C content of the FGIs and the rest of the genome and the presence of mobile genetic elements provide evidence of horizontal gene transfer occurring within the FGI loci. These loci therefore encode highly variable flagellin glycan structures, with distinct sugar backbones, heavily substituted with formyl, methyl, acetyl, lipoyl and amino groups. Additionally, an *N-*lysine methylase, FliB, previously identified only in the enterobacterial pathogen *Salmonella enterica*, is relatively common among several distinct taxa within the family. These flagellin methylase island loci (FMIs), in contrast to the FGI loci, appear to be stably maintained within these diverse lineages.

**Conclusions:**

The prevalence and versatility of flagellin modification loci, both glycosylation and methylation loci, suggests they play important biological roles among the *Enterobacteriaceae*.

**Electronic supplementary material:**

The online version of this article (doi:10.1186/s12864-016-2735-x) contains supplementary material, which is available to authorized users.

## Background

Members of the family *Enterobacteriaceae* are globally distributed and include some of the most important plant, animal and clinical pathogens [1]. As a result, this family is subject to a strong sequencing bias, representing ~ 16 % of all the currently sequenced bacterial genomes [2]. The family also includes the model organism *Escherichia coli* K-12 and the model for bacterial pathogenesis *Salmonella enterica* serovar Typhimurium [3, 4]. As these organisms can be easily manipulated and studied using currently available molecular genetic tools, they have been the basis of invaluable contributions to our current understanding of bacterial physiology, genetics and biology, including metabolism, replication, cell structure and organisation, evolution, metabolism, and swimming motility [3, 4]. Swimming motility in the *Enterobacteriaceae* is by means of peritrichous flagella; filamentous structures extruding from the surface of the bacterial cell [5, 6]. These flagella are comprised of four main components; a motor, basal body, hook and filament. The latter is composed of up to 20,000 copies of a single protein, flagellin (FliC) [6]. The genetic basis of flagellum biosynthesis has been determined in both *E. coli* and *S. enterica* and involves ~50 genes, which are clustered in three distinct chromosomal regions. Gene cluster III incorporates the *fliC* gene coding for flagellin, as well as the flagellum capping protein (*fliD*) and the flagellum-specific sigma factor (*fliA*) [7].

As flagellar motility facilitates the rapid movement of bacterial cells, it is pivotal in allowing bacteria to move towards nutrient-rich environments and away from detrimental niches [7, 8]. In pathogenic microorganisms, flagellar motility plays a role in colonisation, adhesion and biofilm formation, and as such the flagellum is considered as a virulence factor in both plant and animal pathogens [5]. However, the surface domains of the flagellin protein are highly immunogenic, and are recognized by host receptors, triggering innate and adaptive immune responses, both locally and systemically [5, 9, 10]. Motile bacterial pathogens have evolved several means to counteract flagellin recognition. For example, *S. enterica* and some strains of *E. coli* can alternatively express two antigenically distinct flagellin genes in a process known as phase variation [11, 12]. Other pathogens attach sugar chains to the flagellin protein, resulting in a flagellin with distinct antigenic properties, in a process termed post-translational flagellin glycosylation [13]. This phenomenon has been observed in a number of Gram-negative bacteria, including the human and animal pathogens *Campylobacter*, *Aeromonas* and *Pseudomonas aeruginosa*, as well as the phytopathogen *Pseudomonas syringae* [13–16]. The genes responsible for flagellin glycosylation usually occur adjacent to the flagellin gene(s) and often include sugar biosynthetic genes as well as glycosyltransferases [17]. The flagellin glycosylation loci are, however, highly variable, even among strains of the same species, resulting in highly distinct glycan structures. As such, a number of additional functions have been ascribed to flagellin glycosylation, particularly in pathogenic microorganisms, including surface recognition, adhesion, biofilm formation, mimicry of host cell surface glycans and virulence [13, 17, 18].

To date, flagellin glycosylation has not been described in the peritrichously flagellated *Enterobacteriaceae*, despite the extensive body of research on this family. However, a methylase FliB has been described in *S. enterica*, which is involved in the posttranslational modification of the flagellin protein in this bacterium, with a predicted role in swarming motility and/or virulence [19, 20]. Here, by means of comparative genomic approaches, we have demonstrated that flagellin glycosylation loci are relatively common among the *Enterobacteriaceae*. The extensive variability of these loci suggests that they code for highly variable glycans. Furthermore, we show that FliB orthologs are encoded on the genomes of a number of other enterobacterial taxa. Flagellin ornamentation with glycans and methyl groups therefore appears to be relatively common and highly variable among the *Enterobacteriaceae* and we postulate the possible functions associated with this characteristic.

## Results and discussion

### Flagellin ornamentation loci are widespread among the *Enterobacteriaceae*

**Fig. 1:**
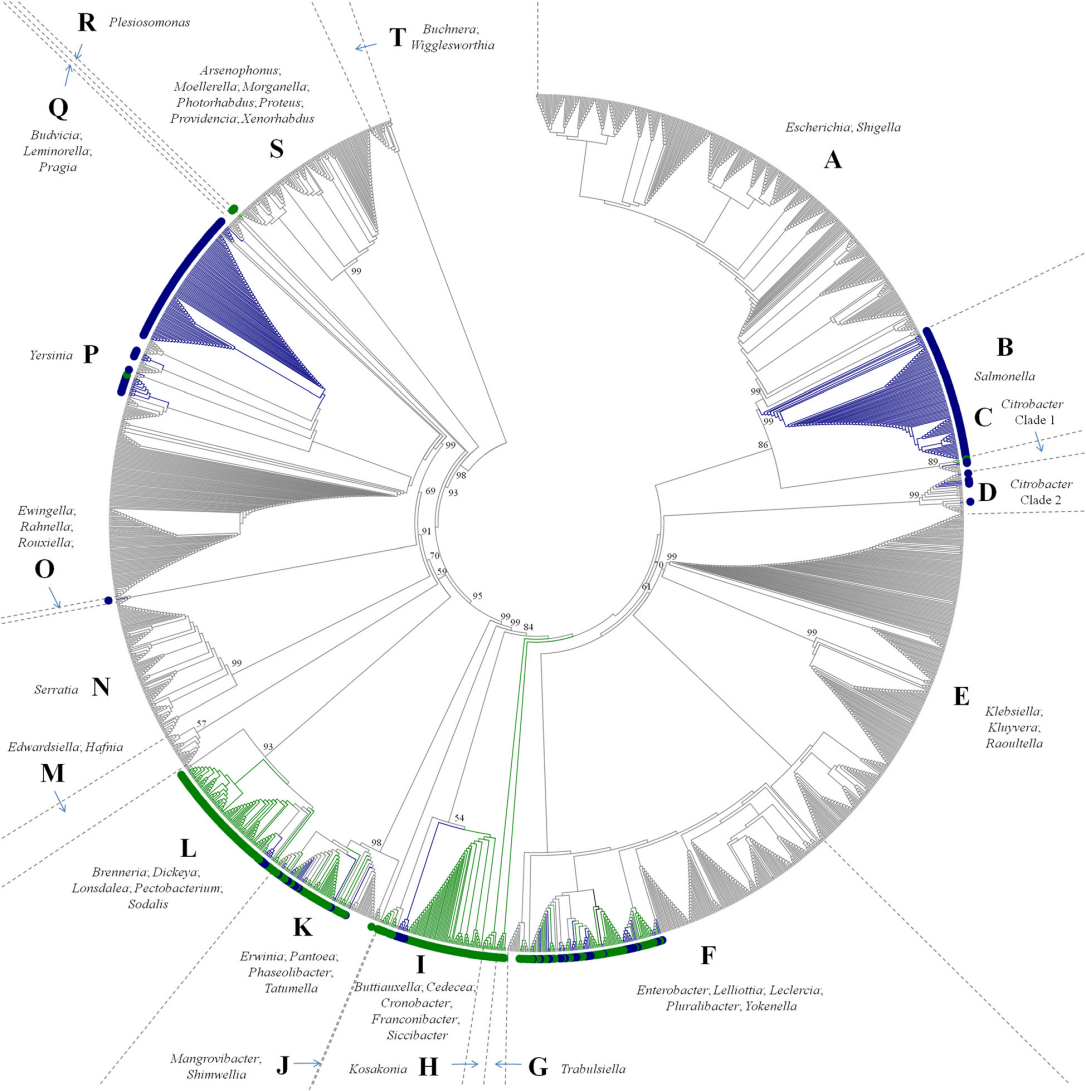
Family-wide distribution of FGI and FMI loci. A circularized, topology-only neighbour-joining phylogeny was constructed on the basis of the concatenated amino acid sequences of the house-keeping markers GyrB, InfB, RecA and RpoB. Bootstrap analysis was performed (*n* = 100) and bootstrap values above 50 % are shown for the major clades. The strains were incorporated in twenty deeper-branching clades (A-T). FGI^+^ *Enterobacteriaceae* are indicated by green dots and branch lines, while FMI^+^ strains are indicated in blue

The complete and draft genomes of 2,000 strains belonging to the family *Enterobacteriaceae*, encompassing 50 distinct genera, were screened for the presence of inserts within their flagellin (*fliDC*/*AZ*) loci (Additional file 1: Table S1). Inserts were located between the *fliC* and *fliA* genes in 631 of the strains. Of these, 307 (15.4 %) contained hallmarks of flagellin glycosylation islands (FGIs), including genes coding for glycosyltransferases and sugar biosynthetic enzymes (Additional file 1: Table S2). A further 302 (15.1 %) strains contained methyltransferases but no other FGI loci genes, and these inserts were termed Flagellin Methylation Islands (FMI) (Additional file 1: Table S3). The majority of the FMI loci (296 strains) encode orthologs of the *N*-lysine methylase FliB, which was first observed in *Salmonella* over 50 years ago [21, 22]. A distinct S-adenosylmethionine-dependent methyltransferase (*smtA*) is inserted in the *fliDC*/*AZ* loci of six *Pectobacterium wasabiae* strains (FMI2). It should be noted that a substantial number of the strains (239 strains) included in the genomic screening of the family lack the *fliDCAZ* loci and are not capable of motility by means of peritrichous flagella, including *Buchnera* spp., *Raoultella* spp., *Wigglesworthia* spp., *Phaseolibacter flectens*, *Serratia symbiotica* and the majority of *Klebsiella* spp.. Thus, when considering only the peritrichously flagellate *Enterobacteriaceae*, 17.4 and 17.1 % of the analyzed strains can be considered to contain FGI and FMI loci, respectively. A neighbour-joining phylogeny was constructed for the 2,000 *Enterobacteriaceae* on the basis of the concatenated amino acid sequences for the house-keeping markers GyrB, InfB and RpoB. This phylogeny (Fig. 1) shows that flagellin ornamentation loci occur in eleven of the eighteen distinct deep-branching clades, with FGI^+^ and FMI^+^ strains occurring in nine and seven of the clades, respectively. The majority of these clades incorporate both FGI^+^ and FMI^−^ strains, while FGI^+^ strains are found exclusively in the deep-branching clades G, H, I and R, and deep-branching clades B, D and O are occupied by FMI^+^ strains only (Fig. 1). At the genus level, FGIs are found in twenty of the fifty sampled genera, while nine genera contain FMI loci (Table 1). Three genera, *Ewingella* (1 strain), *Franconibacter* (8 strains) and *Salmonella* (103 strains) are exclusively comprised of FMI^+^ strains, while eleven genera incorporate only FGI^+^ strains. For three further genera, *Brenneria* (2 strains), *Leclercia* (2 strains) and *Tatumella* (4 strains), only a single strains was FMI^+^ (Table 1). Seven genera incorporated both FGI^+^ and FMI^+^ strains. In general, the presence of either type of flagellin ornamentation locus appeared mutually exclusive, with the exception of two strains, *Pantoea agglomerans* GB1 and *Pantoea vagans* C9-1, where the flagellin ornamentation loci contained both a *fliB* gene, as well as a gene coding for a GT25 family glycosyltransferase which may be involved in flagellin glycosylation.

**Table 1:** Prevalence and proportions of FGIs and FMIs for each of the sampled genera of the *Enterobacteriaceae*

Genus	Clade	Strains	Species	FGI	FGI types	FMI
*Arsenophonus*	S	3	1	0 (0 %)	-	0 (0 %)
*Brenneria*	L	2	2	**1 (50 %)**	1	0 (0 %)
*Buchnera*	T	15	1	0 (0 %)	-	0 (0 %)
*Budvicia*	Q	1	1	0 (0 %)	-	0 (0 %)
*Buttiauxella*	I	2	1	0 (0 %)	-	0 (0 %)
*Cedecea*	I	6	3	**6 (100 %)**	2	0 (0 %)
*Citrobacter*	C/D	43	10	**2 (5 %)**	1	**8 (19 %)**
*Cronobacter*	I	60	6	**60 (100 %)**	3	0 (0 %)
*Dickeya*	L	44	9	**44 (100 %)**	4	0 (0 %)
*Edwardsiella*	M	20	5	0 (0 %)	-	0 (0 %)
*Enterobacter*	F	252	14	**66 (26 %)**	10	**35 (14 %)**
*Erwinia*	K	32	11	**8 (25 %)**	4	**1 (3 %)**
*Escherichia*	A	136	7	0 (0 %)	-	0 (0 %)
*Ewingella*	O	1	1	0 (0 %)	-	**1 (100 %)**
*Franconibacter*	I	8	2	0 (0 %)	-	**8 (100 %)**
*Hafnia*	M	8	2	0 (0 %)	-	0 (0 %)
*Klebsiella*	E	279	5	0 (0 %)	-	0 (0 %)
*Kluyvera*	E	2	2	0 (0 %)	-	0 (0 %)
*Kosakonia*	H	11	8	**11 (100 %)**	5	0 (0 %)
*Leclercia*	F	2	1	**1 (50 %)**	1	0 (0 %)
*Lelliottia*	F	1	1	**1 (100 %)**	1	0 (0 %)
*Leminorella*	Q	1	1	0 (0 %)	-	0 (0 %)
*Lonsdalea*	L	4	2	**4 (100 %)**	2	0 (0 %)
*Mangrovibacter*	J	1	1	**1 (100 %)**	1	0 (0 %)
*Moellerella*	S	1	1	0 (0 %)	-	0 (0 %)
*Morganella*	S	14	1	0 (0 %)	-	0 (0 %)
*Pantoea*	K	56	16	**37 (66 %)**	8	**12 (21 %)**
*Pectobacterium*	L	52	5	**45 (87 %)**	11	**6 (12 %)**
*Phaseolibacter*	K	1	1	0 (0 %)	-	0 (0 %)
*Photorhabdus*	S	14	4	0 (0 %)	-	0 (0 %)
*Plesiomonas*	R	3	1	**3 (100 %)**	3	0 (0 %)
*Pluralibacter*	F	10	1	0 (0 %)	-	0 (0 %)
*Pragia*	Q	1	1	0 (0 %)	-	0 (0 %)
*Proteus*	S	43	4	0 (0 %)	-	0 (0 %)
*Providencia*	S	22	6	0 (0 %)	-	0 (0 %)
*Rahnella*	O	4	1	0 (0 %)	-	0 (0 %)
*Raoultella*	E	11	2	0 (0 %)	-	0 (0 %)
*Rouxiella*	O	1	1	0 (0 %)	-	0 (0 %)
*Salmonella*	B	103	2	0 (0 %)	-	**103 (100 %)**
*Serratia*	N	111	12	0 (0 %)	-	0 (0 %)
*Shigella*	A	243	4	0 (0 %)	-	0 (0 %)
*Shimwellia*	J	1	1	0 (0 %)	-	0 (0 %)
*Siccibacter*	I	4	2	**4 (100 %)**	1	0 (0 %)
*Sodalis*	L	3	3	0 (0 %)	-	0 (0 %)
*Tatumella*	K	4	3	**1 (25 %)**	1	0 (0 %)
*Trabulsiella*	G	8	2	**8 (100 %)**	1	0 (0 %)
*Wigglesworthia*	T	4	2	0 (0 %)	-	0 (0 %)
*Xenorhabdus*	S	25	8	0 (0 %)	-	0 (0 %)
*Yersinia*	P	324	19	**1 (0.3 %)**	1	**128 (40 %)**
*Yokenella*	F	3	2	**3 (100 %)**	2	0 (0 %)

The clade in the family-wide phylogeny (Fig. 1) in which they occur is indicated, as well as the number of different FGI types for each FGI^+^ genus

The number of strains and % of the total strains analysed for each genus containing FGIs and FMIs are indicated in bold

### Enterobacterial FGIs contain hallmarks of horizontal gene transfer, while the FMIs are stably maintained in distinct enterobacterial lineages

The enterobacterial flagellin glycosylation islands range in size from ~2.4 kb (*Pantoea eucalypti* 299R) to ~ 23 kb (*Pantoea ananatis* B1–9) and encode between one and eighteen proteins. On average the FGIs have a G + C content 5.2 % (range -17.7 to +4.0 %) below the genomic average, suggestive of recent horizontal acquisition (Additional file 1: Table S2). Further evidence for HGT is provided by the presence of twenty-one transposase genes, with between zero and six (*Kosakonia* sp. CAV1151) distinct transposases encoded within the FGIs. Two distinct HNH family endonucleases (*edn1* – *Pantoea* and *Erwinia* spp.; *edn2* – *Pectobacterium carotovorum* BC S2) were also identified (Additional file 1: Table S4). These homing endonucleases have been identified in bacteriophages, bacteria, archaea and eukarya and drive their own integration and replication in a host genome [23]. Several FGI^−^/FMI^−^ enterobacteria contain inserts within the *fliDC*/*AZ* locus, with distinct putative functions not related to flagellin ornamentation (Additional file 2: Figure S1; Additional file 1: Table S5). These code for proteins involved in amino acid and phosphate sugar transport and metabolism, fimbrial biogenesis and transposition. Furthermore, distinct prophages are incorporated in this region in some FGI^−^/FMI^−^ enterobacteria. The flagellin locus of the FGI^+^ strains *Erwinia tracheiphila* BuffGH and PSU-1 also incorporate an *Escherichia* phage D108-like prophage upstream of the FGI loci. In the former strain, this prophage is also integrated in two further locations on the chromosome, suggesting that the FGI and prophage incorporation in the flagellin locus result from two distinct integration events. A Enterobacteria P88-like prophage is also incorporated in the flagellin locus of the FMI^+^ strain *Citrobacter rodentium* ICC168. What role, if any, these prophages may have in flagellin ornamentation or functioning remains to be determined. The presence of a large number of highly diverse FGI and FMI loci and inserts of other functions within the *fliDC/AZ* loci of the *Enterobacteriaceae*, as well as the presence of mobile genetic elements such as transposases, endonucleases and prophages (with predicted roles in HGT), suggests that the region downstream of the *fliC* gene serves as a hot-spot for the integration of foreign DNA. However, no standard signature for integration, such as repetitive elements or tRNA genes, could be observed within the loci.

**Fig. 2:**
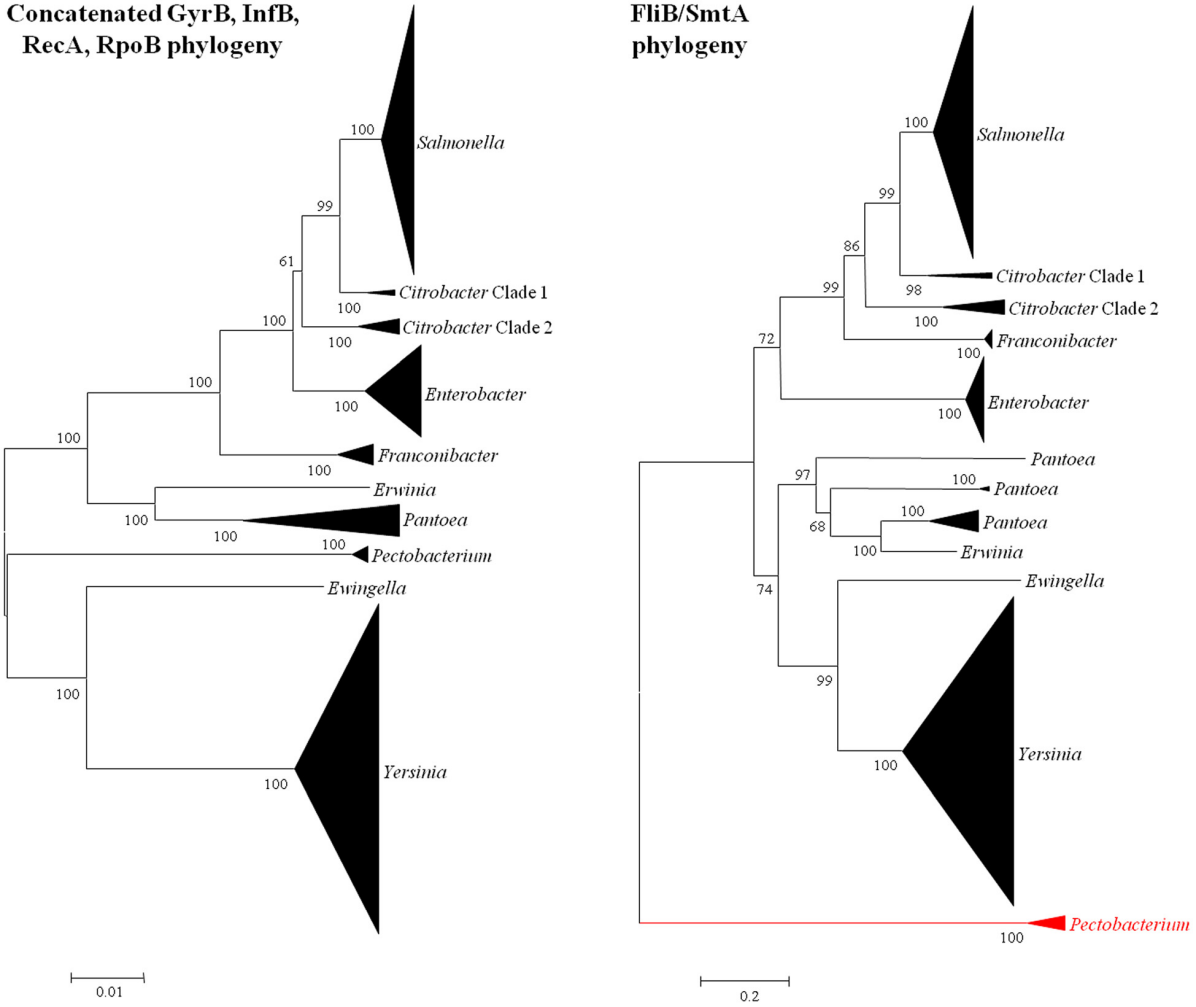
Flagellin methylase phylogeny versus house-keeping marker phylogeny. Neighbour-joining phylogenies were constructed on the basis of alignments of the concatenated house-keeping markers GyrB, InfB, RecA and RpoB (*left*) and the flagellin methylases FliB and SmtA (*right*). Bootstrap analyses were performed (*n* = 1,000) and bootstrap support values above 50 % are shown. The red branch indicates the distinct methylase (SmtA) encoded in the FMI locus of *Pectobacterium wasabiae*

As observed for the FGIs, the FMIs, which range in size from 866 to 3,358 nucleotides in size, have an average G + C content 7.2 % (range -12.9 to -1.6) below the genomic average, suggesting that they have also been derived from recent HGT events (Additional file 1: Table S3). It should, however, be noted that the *fliB* gene is universal in several monophylogenetic lineages, including those of *Salmonella enterica* (100 strains) and *Yersinia enterocolitica* (100 strains) (Fig. 1). This would indicate that, at least in these species, the FMI is ancestral and vertically maintained throughout the species. A neighbour-joining phylogeny of the FliB/SmtA protein sequences of all FMI^+^*Enterobacteriaceae* (Fig. 2) shows good congruence with the phylogeny on the basis of the concatenated house-keeping markers GyrB, InfB and RpoB. *Pectobacterium wasabiae*, which encodes a different methyltransferase (SmtA) from the other FMI^+^ taxa (FliB), forms a distinct clade. Thus, in contrast to the FGIs, the FMIs appear to be a more ancient trait, which is stably maintained within subpopulations of the family. In addition to the *fliB* gene, the FMI loci of the *S. enterica* serovar Typhimurium strains incorporate an insertion element, IS*200*, which has been used as a genetic marker for the differentiation of this serovar [21, 24]. The presence of this IS, however, does not affect the clustering of the serovar Typhimurium strains with the other *S. enterica* serovars, suggesting a distinct origin of the *fliB* and IS*200* genetic elements. Similarly, the *fliB* gene in twelve *Pantoea* and one *Erwinia* strain are flanked by the HNH endonuclease gene *edn1*, which is likewise found in the FGI^+^*Pantoea* and *Erwinia* strains, suggesting *fliB* and *edn1* are likely derived through two distinct HGT events.

### Enterobacterial flagellin glycosylation islands are highly versatile and are poorly conserved among and within species

**Fig. 3:**
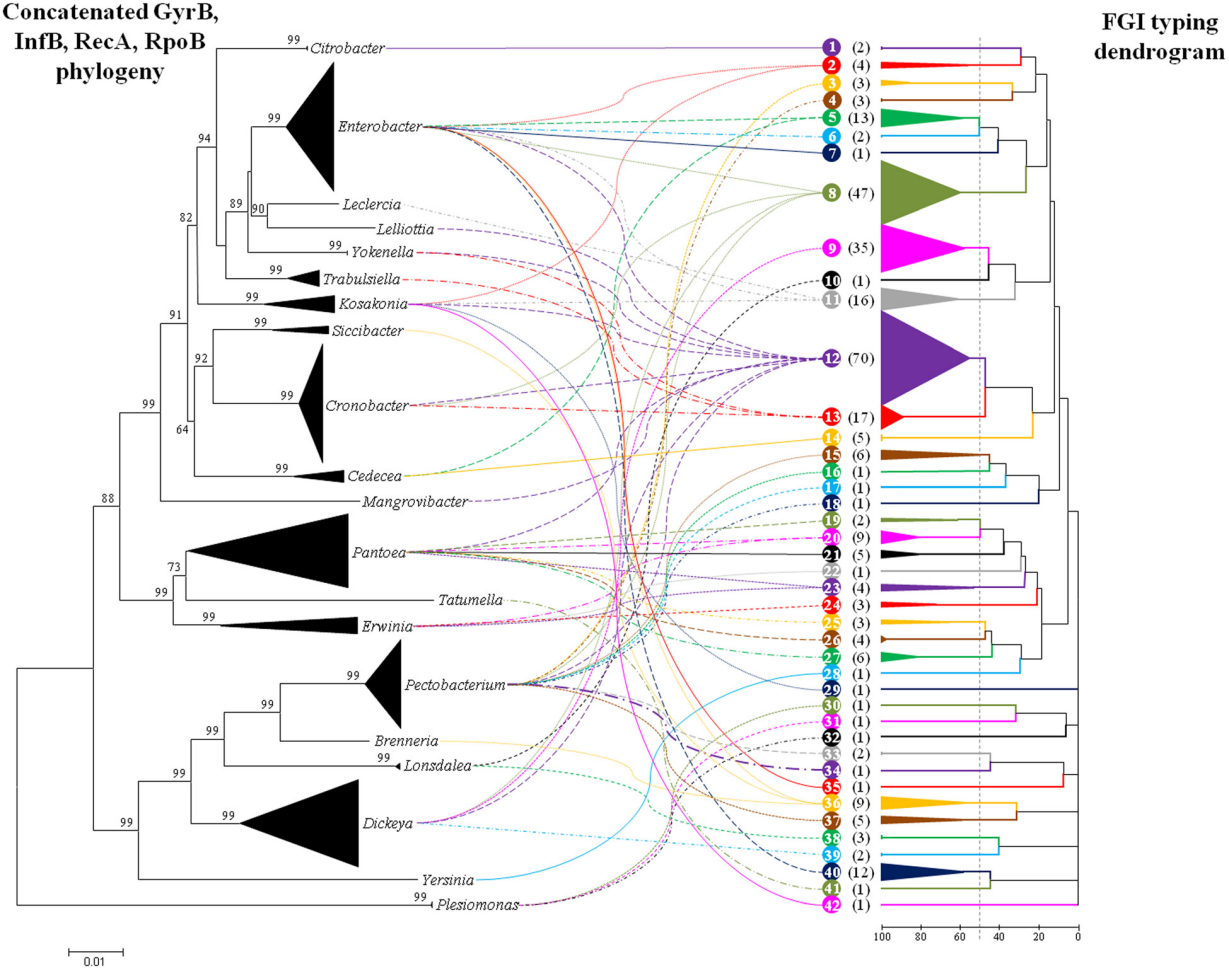
Flagellin glycosylation typing dendrogram. A typing dendrogram was constructed on the basis of a distance matrix representing the presence/absence of orthologs of each of the 218 distinct proteins encoded within the FGIs (*right*). FGI types were distinguished on the basis of 50 % distance cut-off values. The FGI typing dendrogram was compared against a neighbour-joining phylogeny on the basis of the amino acid sequences of the house-keeping markers GyrB, InfB, RecA and RpoB (*left*) for all the FGI^+^ *Enterobacteriaceaea*. Bootstrap analyses were performed for the latter phylogeny and bootstrap values above 50 % are shown

The protein sequence sets encoded within the FGIs were compared and orthologs were identified, where orthologs were assigned on the basis of amino acid identity and sequence coverage thresholds of > 50 and 70 %, respectively. A total of 218 distinct proteins were identified within the enterobacterial FGIs, with no single protein shared among all 307 FGI^+^ taxa. A FGI typing scheme was developed on the basis of the presence/absence of protein orthologs, with the resulting distance matrix being utilised to generate a dendrogram representing all 307 FGI^+^ strain protein datasets. Using a 50 % similarity threshold (i.e. >50 % of the proteins encoded in the FGI of one strain had orthologs in the FGI of a compared strain), 42 distinct types of FGIs could be distinguished (Fig. 3, Additional file 3: Figure S2). This value increased substantially when higher cut-off values were applied, where 70 % similarity and 100 % identity threshold values yielded 59 and 88 distinct FGI types, respectively. The number of FGI types, regardless of the cut-off values applied, was consistently higher than the number of FGI^+^ genera, indicating that more than one type of FGI type occurs for some genera. For example, the forty-five FGI^+^*Pectobacterium* strains could be classified into eleven distinct FGI types, eight FGI types could be distinguished for the thirty-seven FGI^+^*Pantoea* spp., while three *Plesiomonas shigelloides* isolates had three distinct FGI types. On the other hand, monotypic FGIs could be observed for several genera, including *Citrobacter*, *Siccibacter* and *Trabsuliella*, with two, four and eight strains in these genera incorporated in this study (Table 1). Nevertheless, there is evidence of extensive horizontal exchange of the FGI elements. For example, FGI type 12 strains include members of the genera *Dickeya, Enterobacter, Kosakonia, Lelliottia, Mangrovibacter, Pantoea, Pectobacterium* and *Yokenella*, falling within three deeper branching clades of the house-keeping marker phylogeny (Fig. 1).

**Fig. 4:**
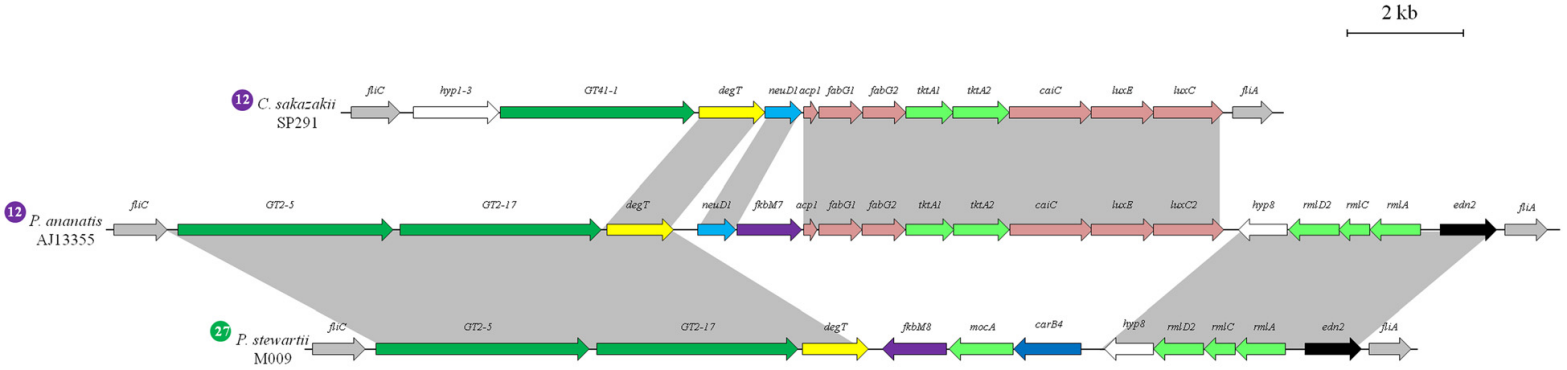
Alignment of the type 12 FGI loci of *P. ananatis* AJ1335 and *C. sakazakii* SP291 and the type 27 FGI locus of *P. stewartii* M009. Glycosyltransferase and sugar biosynthetic genes are indicated by dark and light green arrows, respectively. Formyltransferases, methyltransferases, acetyltransferases and aminotransferases are encoded by genes represented by dark blue, purple, light blue and yellow arrows, respectively. Pink arrows indicates genes involved in fatty acid biosynthesis. Flanking genes are indicated by grey arrows, genes coding for hypothetical proteins by white arrows and black arrows indicate endonuclease (*edn2*) genes. The grey blocks indicate the regions of homology between the compared strains

Comparison of the type 12 FGI locus of *P. ananatis* AJ13355 with that of the type 12 FGI strain *Cronobacter sakazakii* SP291, shows that they share a gene cluster coding for the biosynthesis of fatty acid chains which may lipoylate the glycan sugars. Similarly, a rhamnose biosynthetic cluster in the *P. ananatis* AJ13355 FGI is also found in the type 27 FGI of *Pantoea stewartii* M009, but not in the *C. sakazakii* SP291 FGI locus (Fig. 4). This suggests that the FGIs are not necessarily transferred *en bloc*, but instead, have developed through the incorporation of gene clusters through several distinct horizontal gene transfer events. This would further add to the structural complexity of the glycan encoded by the FGI^+^ strains.

### Enterobacterial FGIs code for glycans with a wide range of backbone sugars

Glycosyltransferases catalyse the transfer of a saccharide onto a sugar or non-sugar acceptor, and form an integral part of the post-translational protein glycosylation machinery [25]. Using the orthology cut-off values of 50 % amino acid identity over 70 % of the shared sequence, and BlastP analysis against the NCBI non-redundant protein database [26], forty-three distinct glycosyltransferases (GTs) could be distinguished, with between one and three GTs encoded per FGI. BlastP comparison against the Carbohydrate-Active enzymes (CAZy) database using the dbCAN pipeline [27, 28], classified thirty-one of these as belonging to the GT2 family, while one and eleven glycosyltransferases, respectively, were classified in the GT25 and GT41 families [25]. The presence of orthologs of GT enzymes of each family appear to be mutually exclusive, with each FGI^+^ strains only containing GTs belonging to a single family. The GT2 enzymes transfer a broad range of saccharides, including *N*-acetyl-glucosamine, mannose, glucose, galactose, rhamnose and their derivatives [25]. Thus the type of sugars incorporated in the flagellin glycans cannot be determined on the basis of the GT2 enzyme. The GT25 family, which is found in the type 21 FGIs of five *Pantoea* strains, includes glucosyltransferases and galactosyltransferase and is involved in lipooligosaccharide biosynthesis in *Moraxella catarrhalis*, *Neisseria gonorrhea* and *Haemophilus influenzae* [29]. Similarly, the GT41 family includes peptide *N-*β-glucosyltransferases and peptide β-*N*-acetylglucosaminyltransferases. The eleven distinct FGI GT41 enzymes all incorporate a conserved SPINDLY family *O*-linked *N*-acetylglucosamine transferase domain (COG3914). The presence of this domain suggests that the flagellin of 198 FGI^+^ strains, which occur in fourteen of the forty-two FGI types containing GT41 orthologs, are glycosylated with *N*-acetylglucosamine derivatives. However, considering the low level of homology among the FGI GT41 family proteins (43.6 % average amino acid identity; range 7 to 100 %) the possibility of wider transferase specificity exists.

**Fig. 5:**
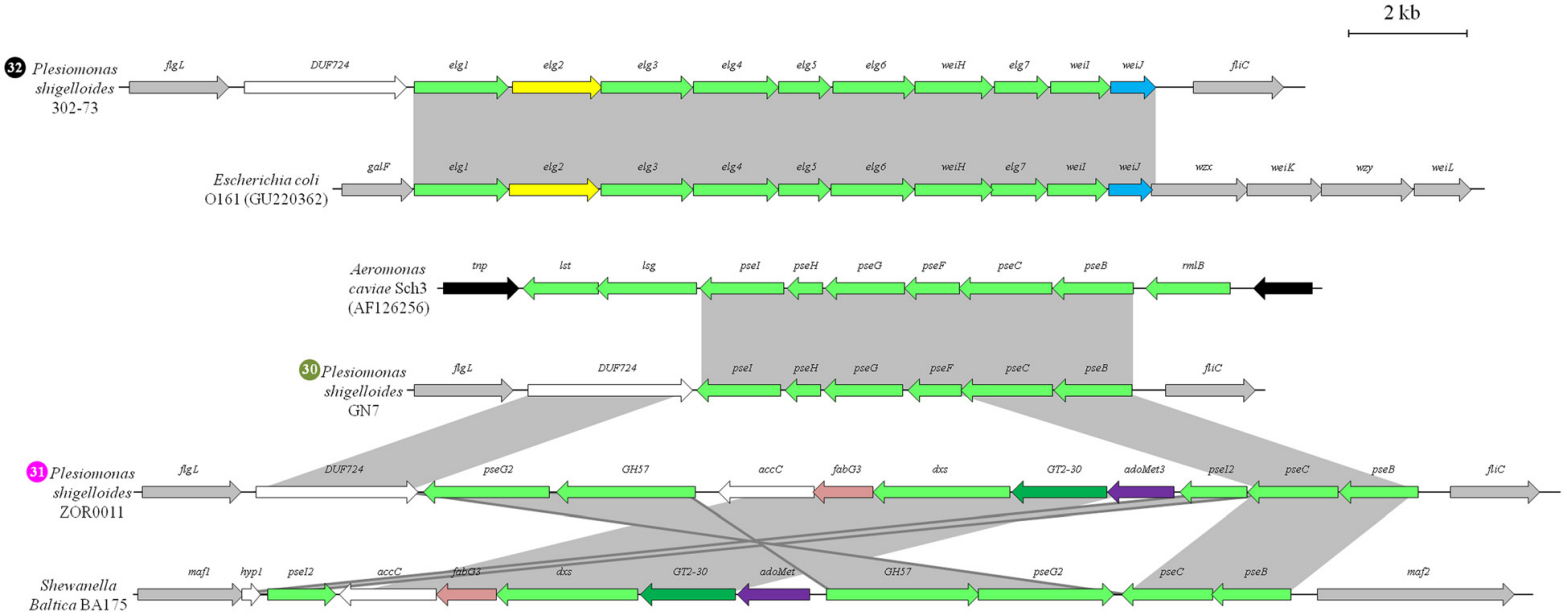
Alignment of the FGI loci of three distinct *Plesiomonas shigelloides* strains with homologous loci in other bacteria. The sugar biosynthetic genes are indicated by light green arrows, while the glycosyltransferases are represented by dark green arrows. Putative fatty acid biosynthesis, acetyltransferase, aminotransferase and methyltransferase genes are depicted by pink, light blue, yellow and purple arrows, respectively. The black arrows indicate transposase genes, while flanking genes are coloured in grey. The grey blocks indicate the regions of homology between the compared strains

The presence of genes involved in the biosynthesis of several distinct sugar moieties provides an insight into the flagellin glycan backbone in several FGI^+^ enterobacteria. Most common among the sugar biosynthetic proteins are those involved in rhamnose biosynthesis, present in 28 FGI^+^ strains and six of the forty-two FGI types. The four genes required for rhamnose biosynthesis, *rmlABCD*, are also found in the capsular polysaccharide and LPS O-antigen biosynthetic loci in a wide range of *Enterobacteriaceae* [30, 31]. Among the FGI^+^ strains only one strain, *Erwinia billingiae* M043b (FGI type 22), encodes the full complement of Rml proteins, while all other strains lack the *rmlB* gene coding for dTDP-D-glucose 4,6 dehydratase, which catalyzes the second step in rhamnose biosynthesis, dehydration of dTDP-D-glucose to dTDP-4-keto-6-deoxy-D-glucose [30]. Copies of *rmlB* are, however, found elsewhere on the genome, including in the O-antigen biosynthetic clusters, and these may complement this function in flagellin glycan biosynthesis. Other FGIs incorporate genes coding for enzymes involved in the biosynthesis of CDP-4-keto-6-deoxy-D-glucose (4 strains, FGI type 23), galactofuranose (2 strains; FGI type 39), neuraminic acid (1 strain; FGI type 16), a dTDP-6-deoxy-hex-4-ulose derivative (1 strain; FGI type 18), the sugar acid UDP-D-gluconate (1 strain; FGI type 36) and the pentose sugar alcohol ribitol (2 strains; FGI type 12). The diversity of sugar biosynthetic pathways incorporated in the FGIs is further highlighted by the three *Plesiomonas shigelloides* strains included in this study, where genes for the biosynthesis of three distinct sialic acid derivatives are present. The type 32 FGI of *P. shigelloides* 302—73 codes for ten proteins sharing 63.9 % average amino acid identity with those involved in the production of legionaminic acid in the O-antigen of *Escherichia coli* O161 [32], while loci for the biosynthesis of two distinct neuraminic/pseudaminic acid derivatives occur in the type 30 and 31 FGIs of *P. shigelloides* GN7 and ZOR011, respectively (Fig. 5).

### Formylation, methylation, acetylation and lipoylation add to the complexity of the enterobacterial flagellin glycans

The flagellin glycan chains of some bacterial taxa are extensively modified or substituted with methyl, formyl, acetyl or amino groups. For example, an acetamidino group is incorporated in the flagellum *N*-glycan of *Methanococcus maripaludis* [33], while formyl and amino substitutions have been observed on sugars in the flagellin glycan of *Pseudomonas aeruginosa* [14]. An acetyltransferase encoded in the flagellar region of *Xanthomonas oryzae* is essential for flagellin glycosylation, as well as playing roles in motility, exopolysaccharide production and biofilm formation [34]. The most common modification of the flagellin glycan among the *Enterobacteriaceae* appears to be amination, with aminotransferases found in the FGIs of 242/307 FGI^+^ strains. These include UDP-4-keto-6-deoxy-N-acetylglucosamine-4-aminotransferases (PseC; 2 strains in two FGI types) and three distinct types of DegT/DnrJ/EryC1/StrS family aminotransferases (DegT1-2 and FdtB; 242 strains in twenty-three FGI types). Furthermore, methylation may occur in 235/307 FGI^+^ strains. Methyltransferases belonging to three distinct families are encoded within the FGIs; S-adenosylmethionine-dependent methyltransferases (AdoMet1–14; 73 strains in fourteen FGI types), FkbM family methyltransferases (FkbM1–10; 67 strains in eight FGI types) and a demethylmenquinone family methyltransferase (Mtf; 1 strain in one FGI type). With the exception of eight GT41–11 enzymes, all remaining GT41 glycosyltransferases (194 strains in fifteen FGI types) as well as the GT2–30 glycosyltransferase of *P. shiggeloides* ZOR0011 also contain methyltransferase domains (AdoMet_MTases, cd02440). Two distinct types of proteins involved in O-acetylation and two involved in N-acetylation of a sugar moiety are encoded in the enterobacterial FGIs. These include sialic acid O-acetyltransferase (NeuD1–5; 92 strains in five FGI types), and maltose family O-acetyltransferase (WbbJ1–2; 5 strain in three FGI types), UDP-4-amino-4,6-dideoxy-N-acetyl-beta-L-altrosamine N-acetyltransferase (PseH; 1 strain in one FGI type), and an N-acetyltransferase of unknown specificity (Nat1–5; 60 strains in two FGI types). Furthermore, formyltransferases (fmt1–4) are encoded in the FGIs of nine strains, belonging to four distinct FGI types. Some strains have identical complements of glycosyltransferase and sugar biosynthetic proteins, but differ in terms of these sugar modification enzymes. For example, the FGI type 9 strains *Dickeya* sp. nov 3 (S1) and sp. nov 3 (2B12) are distinguished only by the presence of a FkbM family (FkbM4) methyltransferase in the former, with an orthologs of this protein not encoded within the FGI of the latter. While the FGI of the Type 15 FGI strain *Pectobacterium carotovorum* CFIA1001 encodes a formyltransferase (Fmt3), *P. carovotorum* ICMP 5702 encodes an O-acetyltransferase (WbbJ2) within its type 15 FGI. It can thus be expected that these modification enzymes add to the versatility and complexity of the flagellin glycan structure.

The FGIs of a large number of FGI^+^ strains also encode several proteins involved in fatty acid synthesis (FAS), including acyl carrier proteins (Acp1–2; 72 strains in three FGI types), an acyl-protein synthetase (LuxE; 73 strains in three FGI types), acyl-CoA reductases (LuxC1–4; 80 strains in four FGI types), fatty acid CoA ligases (CaiC1–2; 71 strains plus two partial genes in two FGI types), 3-ketoacyl-(acyl-carrier protein) reductases (FabG; 71 strains in two FGI types) and beta-hydroxyacyl-ACP dehydratases (FabZ; one strain in one FGI type). These form part of and share orthology with proteins involved in *de novo* biosynthesis of fatty acid in *E. coli* [35]. The presence of genes coding FAS enzymes suggests that in some enterobacteria the flagellin glycan may incorporate a liposugar component.

### Flagellin modifications in enterobacteria: not just for looking pretty?

A wide range of different functions have been ascribed to flagellin glycosylation. Key functions which have been reported include masking of the immunogenic epitopes on the flagellin protein, thereby avoiding host recognition during infection and evasion of the resulting host defense response, immunomodulation, surface recognition and attachment, biofilm formation, protection against proteolytic degradation as well as virulence [13, 36, 37].

In several Gram-negative pathogens, including *Campylobacter* and *Aeromonas* spp., as well as the aquatic bacterium *Caulobacter crescentus*, flagellin glycosylation is essential for the assembly and motility function of the flagellum [13]. The vast majority of *Enterobacteriaceae*, regardless of the presence of flagellin ornamentation loci are, however, described as motile. For example, FGI^−^/FMI^−^*Escherichia coli* strains have nevertheless been demonstrated to be motile by means of peritrichous flagella [38]. Deletion of the *fliB* gene also did not affect swimming motility of the FMI^+^*S. enterica* serovar Typhimurium LT2 [19]. It is thus likely that flagellar ornamentation is not required for flagellum synthesis, maintenance or motility and that it may serve alternative functions within the *Enterobacteriaceae*.

The isolation sources of the FGI^+^ and FMI^+^ strains were recorded (Table 2), with the majority of the FGI^+^ enterobacteria being isolated from plant and human hosts. The vast majority of these (85.6 % of the strains from these hosts and 62.5 % of the FGI^+^ strains) were pathogens or of clinical relevance, suggesting that the FGI may play a role in pathogenesis and/or virulence. Given the extensive variability in gene content of the FGIs, even among strains of the same species, the masking of the immunogenic flagellin from detection by the host is a plausible function for the enterobacterial flagellin glycans. This has been observed in several human pathogens, including *Burkholderia cenocepacia* and *Campylobacter* spp., as well as the phytopathogen *Pseudomonas syringae* [15, 16, 39]. The versatile structures of the *Pectobacterium carotovorum* FGI, which belong to nine distinct FGI types, may aid in the infection of the same host plant by *P. carotovorum* strains with different FGI types, without host detection and associated defense responses. Alternatively, flagellin glycosylation may serve as a determinant of host specificity, as observed in *P. syringae* pv. *glycinea*, where the flagellin glycan can induce a hyper-sensitive response in non-host plants [40]. The FGIs are not restricted to pathogens. However, as there is an apparent bias towards the sequencing of plant and animal-pathogenic isolates, the prevalence of FGIs in environmental isolates may be underestimated. These factors, along with the extensive variability of the FGIs and the glycan structures they may encode, would suggest that alternative roles for flagellin glycosylation among the various enterobacterial taxa must be considered.

**Table 2:** Isolation sources of the FGI^+^ and FMI^+^ enterobacterial taxa

Locus	Isolation Source	# strains (% positive strains)	Specific source/relationship with host	#strains (% source)
FGI	Plant	129 (42.0 %)	Pathogen	103 (79.8 %)
			Saprophyte	26 (20.2 %)
	Human	96 (31.3 %)	Clinical	91 (94.8 %)
			Commensal	5 (5.2 %)
	Environmental	55 (17.9 %)	Fresh/Marine water	32 (58.2 %)
			Food	12 (21.8 %)
			Soil	9 (16.4 %)
			Other	2 (3.6 %)
	Animal	12 (3.9 %)	Vertebrate	1 (8.3 %
			Invertebrate	11 (91.7 %)
	Not available	15 (4.9 %)		
FMI	Human	137 (45.4 %)	Clinical	137 (100 %)
	Animal	79 (26.2 %)	Vertebrate	77 (97.5 %)
			Invertebrate	2 (2.5 %)
	Environmental	42 (13.9 %)	Food	24 (57.1 %)
			Soil	7 (16.7 %)
			Fresh/Marine water	4 (9.5 %)
			Other	7 (16.7 %)
	Plant	21 (7.0 %)	Pathogen	7 (33.3 %)
			Saprophyte	14 (66.7 %)
	Not available	23 (7.6 %)		

The specific source and/or relationship with host form which the strains were isolated and the relative proportions for each category of isolation source are shown

The FMIs are mostly observed in the genomes of human and animal isolates, which is probably due to the universal occurrence of this locus in *S. enterica* and *Y. enterocolitica*, where vertebrate hosts serve as a natural reservoir for these bacterial taxa. The genomes of only a limited number of plant-associated bacteria contain an FMI, and these include the distinct flagellin methylation island (FMI2) observed in *P. wasabiae.* The prevalence of the *fliB* among clinical *S. enterica* isolates, and the observation that deletion of this gene did not directly affect swimming motility, have led to the hypothesis that flagellin methylation plays a role in virulence [19]. Recently, a *S. enterica fliB* mutant was shown to be defective in swarming motility [20]. Swarming is the coordinated multi-cellular movement across a semisolid surface and this behaviour plays a role in biofilm formation, colony spread, resistance to antimicrobials, intercellular communication within a swarm, and pathogenesis [20, 41]. Whether flagellin glycosylation plays a role in this phenotype in the other FMI^+^ enterobacteria is unclear. However, swarming motility has been observed in bacteria lacking flagellin methylation islands, such as *E. coli* [42], and thus it is plausible the flagellin methylation may play a role in other phenotypes.

## Conclusions

Despite the huge body of research conducted on the *Enterobacteriaceae*, flagellin glycosylation has to date not been reported for members of this family. Here we provide genomic evidence of flagellin glycosylation islands in a relatively large number of strains, representing a substantial cross section of the taxa in this family. Furthermore, the *fliB* gene, coding for an *N*-lysine methylase is present not only in *S. enterica* strains, but also several other taxa, including *Yersinia*, *Enterobacter*, *Franconibacter* and *Pantoea* spp. This suggests flagellum ornamentation is a relatively common trait among the *Enterobacteriaceae*, in particular among pathogenic strains. This may, however, be as a result of a focus towards genome sequencing of strains of anthrophocentric relevance, and future genome sequencing endeavours may shed further light of the prevalence of flagellin ornamentation loci among environmental strains and species. The extensive genetic variability observed among the flagellin glycosylation islands, in terms of the distinct glycosyltransferases, sugar biosynthetic pathways, even among strains of the same species, suggest they are likely to encode glycans of equally diverse structure and functioning. This may be even further complexed by the variable modification, by means of lipoylation, formylation, acetylation and methylation, of the glycan backbone and/or the flagellin protein.

While genes coding for glycosyltransferases are common features among the enterobacterial FGIs, sugar biosynthetic pathways are encoded within only a limited number of taxa. Furthermore, as exemplified by the rhamnose biosynthetic pathway incorporated in the FGI of several taxa which lacks orthologs of the *rmlB* gene, incomplete or absent pathways may affect the flagellin glycan structure. It is more likely, however, that sugar biosynthetic pathways located elsewhere on the chromosome, or forming part of other glycan biosynthetic processes, such as LPS biosynthesis, may complement this function. As a result, structural characterization of the distinct glycans should be undertaken in order to determine the exact structures of the enterobacterial flagellin glycans. The absence of flagellin ornamentation loci in many *Enterobacteriaceae* which have been demonstrated to possess the capacity of flagellar swimming motility suggests that this trait is not central to the swimming phenotype. Flagellin glycosylation or methylation is thus likely to serve alternative functions among the *Enterobacteriaceae*. Considering the extensive diversity of the FGI and FMI loci, however, we suggest that the role played by these modifications would need to be determined on a case by case basis. The data presented here should thus serve as a primer for further research into the flagellin ornamentation structures as well as the roles that these traits may play in the various enterobacterial taxa in which they occur.

## Methods

### Identification, annotation and characterization of the flagellin ornamentation islands

The genome sequences of 2,000 members of the family *Enterobacteriaceae* were obtained from the NCBI database. The genomes were selected to be representative of the fifty genera for which genome sequences are available. For those species for which more than 100 genomes are available, genome completeness was used as the criterion for subsampling. The FliC and FliA/FlgL proteins of *Escherichia coli* K-12 substr. MG1655 (NCBI Acc. # AAC74990.1 and AAC74989.1) were used in a localized tBlastN analysis with BioEdit v. 7.1.11 [43] to identify the genomic locations of the coding genes in the 2,000 enterobacteria. The *fliC* to *fliA*/*flgL* regions were extracted and the G + C contents of the interior fragments were determined using BioEdit [43]. The regions were structurally annotated using Prokaryotic GeneMark.hmm v. 2 [44] and the protein sequences were functionally annotated by BlastP analysis against the NCBI non-redundant protein database and the NCBI Conserved Domain Database using CD-search [45, 46]. Putative glycosyltransferases were classified according to the Carbohydrate Active enzymes database using the dbCAN Blast tool [27, 28]. Orthologs among the FGI protein datasets were identified using localized BlastP analyses with BioEdit [43]. Orthology was assumed for those proteins which shared >50 % amino acid identity over 70 % of the alignment length.

### Phylogenetic analyses

A phylogeny was constructed for the 2,000 enterobacterial strains using the concatenated amino acid sequences of four house-keeping markers commonly used for delineation of the *Enterobacteriaceae*, Gyrase B (GyrB), translation initiation factor IF-2 (InfB), recombinase A (RecA) and RNA polymerase beta subunit (RpoB). Alignments were generated using the MAFFT v. 7 server and neighbour-joining trees were constructed using the Molecular Evolutionary Genetics Analysis (MEGA) v 5.0.3 software package with the default parameters and bootstrap analysis (*n* = 100 replicates) [47, 48]. Similarly, neighbour-joining phylogenies were constructed for the FGI^+^ and FMI^+^ strains, with bootstrap analyses (*n* = 1,000 replicates). The presence/absence of proteins in the FGI regions were scored, where present orthologs = 1, while absent orthologs = 0. The resultant matrix was used to generate a distance matrix with Bionumerics v 6.6 (Applied Maths N.V., Belgium) using the parameters: absolute values and Pearson’s correlation. This distance matrix was subsequently used to generate an Unweighted Pair Group Method with Arithmetic Mean (UPGMA) dendrogram using Phylip v 3.69 and visualized with MEGA v 5.0.3 [48, 49]. A distance value cut-off value of 50 % was used to discriminate between the FGI types.

## Additional files

Additional file 1: Table S1.
*Enterobacteriaceae* strains analysed in this study. The deep-branching clade to which they belong (Fig. 1), the presence/absence of *fliDCAZ* loci, the NCBI accession numbers of the contigs on which these loci occur, and presence/absence of FGI and FMI loci are indicated. Table S2. Characteristics of FGI^+^ *Enterobacteriaceae* and their flagellin glycosylation islands. The isolation sources and deep-branching clade to which they belong in the *Enterobacteriaceae* phylogeny (Fig. 1) are indicated for each of the FGI^+^ enterobacterial strains. The sizes, G + C contents, G + C deviation from the rest of the genome and number of proteins encoded on each FGI are shown. Table S3. Characteristics of FMI^+^ *Enterobacteriaceae* and their flagellin methylation islands. The isolation sources and deep-branching clade to which they belong in the *Enterobacteriaceae* phylogeny (Fig. 1) are indicated for each of the FMI^+^ enterobacterial strains. The sizes, G + C contents, G + C deviation from the rest of the genome and number of proteins encoded on each FMI are shown. Table S4. Annotations of the proteins encoded on the enterobacterial FGIs. The number of strains and genera in which orthologs of each distinct protein are encoded within the FGIs are indicated, as well as the closest non-enterobacterial Blast hit, obtained by BlastP analysis against the NCBI non-redundant protein database. Orthologs were only considered among the top 500 BLAST hits and for those orthologs with > 30 % amino acid identity to the query protein. The putative function and conserved domains observed after BLAST analyses against the NCBI protein and conserved domain databases are shown. Table S5. Genomics inserts in the *fliDCAZ* loci of FGI^−^/FMI^−^ *Enterobacteriaceae*. The insert size, G + C content, G + C deviation from the rest of the genome, number of proteins encoded and putative functions of the encoded proteins in each insert are shown. (XLSX 234 kb) Additional file 2: Figure S1. Schematic diagrams of inserts within the *fliDCAZ* loci of FGI^−^/FMI^−^ *Enterobacteriaceae*. Flanking genes are indicated by yellow arrows, predicted phage genes by blue arrows, fimbrial biogenesis genes by grey arrows and sugar/amino acid transporter genes by orange arrows. Black arrows indicate predicted transposase or endonuclease genes, while the red arrows indicate genes with disrupted reading frames. The flagellin glycan biosynthetic genes in the FGI^+^ strains *E. tracheiphila* Buff/PSU-1 are indicated by green arrows, upstream of the predicted phage integration site. (TIF 471 kb) Additional file 3: Figure S2. Schematic diagrams of stereotypical flagellin glycosylation islands of the forty-two distinct FGI types. Glycosyltransferase and sugar biosynthetic genes are indicated by dark and light green arrows, respectively. Formyltransferases, methyltransferases, acetyltransferases and aminotransferases are encoded by genes represented by dark blue, purple, light blue and yellow arrows, respectively. Pink arrows indicates genes involved in fatty acid biosynthesis. Flanking genes are indicated by grey arrows, genes coding for hypothetical proteins or involved in functions with no relative known function in flagellin glycosylation by white arrows and black arrows indicate transposes and endonuclease genes. (TIF 3168 kb)

## Abbreviations

CDS: protein coding sequence
FGI: flagellin glycosylation island
FMI: flagellin methylation island
GT: Glycosyltransferase
HGT: horizontal gene transfer
