# Supplementary figures and images for: Flashy flagella: flagellin modification is relatively common and highly versatile among the Enterobacteriaceae
Source: BMC Genomics. 2016 May 20;17:377. doi: 10.1186/s12864-016-2735-x (PMC4875605; doi:10.1186/s12864-016-2735-x)

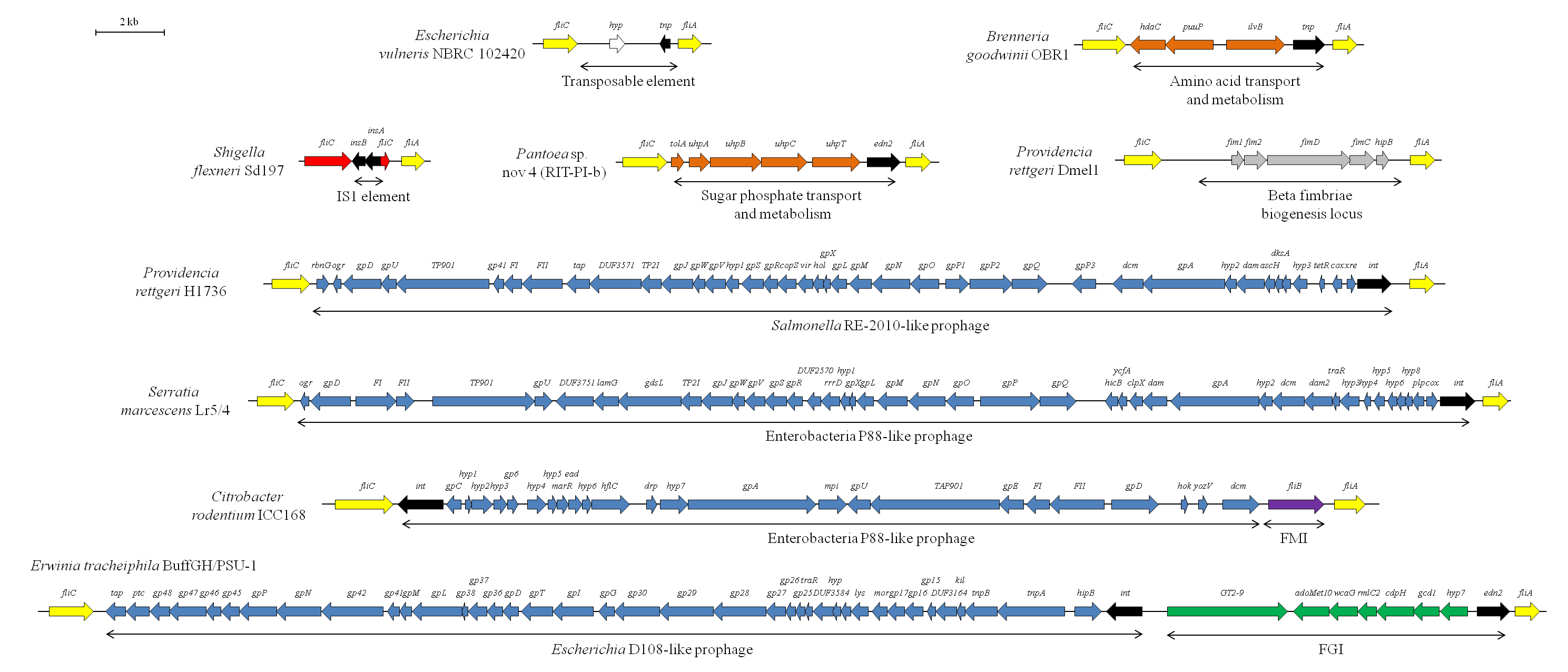

Supplement: Additional file 2: Figure S1. — Schematic diagrams of inserts within the fliDCAZ loci of FGI−/FMI− Enterobacteriaceae. Flanking genes are indicated by yellow arrows, predicted phage genes by blue arrows, fimbrial biogenesis genes by grey arrows and sugar/amino acid transporter genes by orange arrows. Black arrows indicate predicted transposase or endonuclease genes, while the red arrows indicate genes with disrupted reading frames. The flagellin glycan biosynthetic genes in the FGI+ strains E. tracheiphila Buff/PSU-1 are indicated by green arrows, upstream of the predicted phage integration site. (TIF 471 kb) [file 12864_2016_2735_MOESM2_ESM.tif]

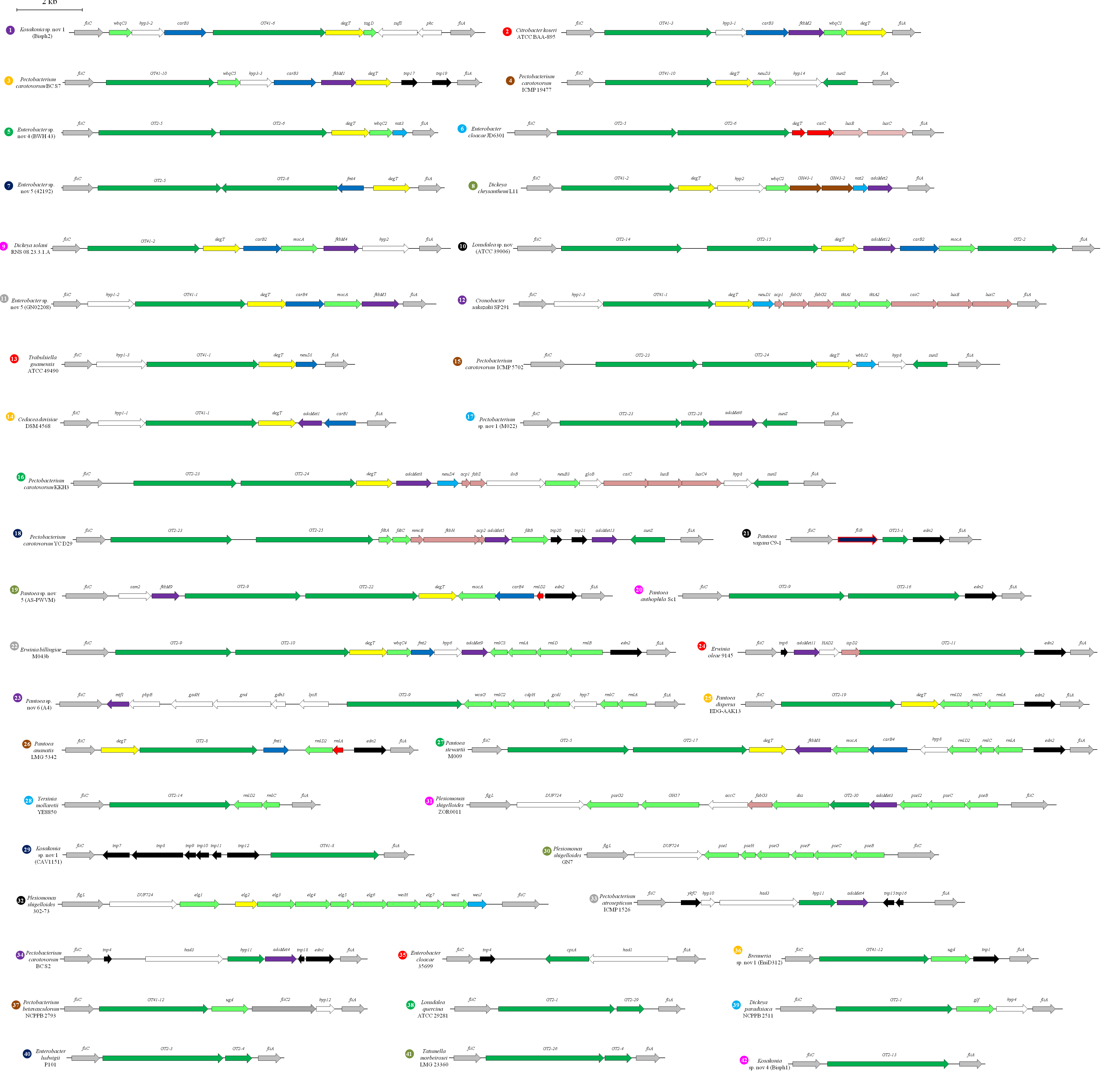

Supplement: Additional file 3: Figure S2. — Schematic diagrams of stereotypical flagellin glycosylation islands of the forty-two distinct FGI types. Glycosyltransferase and sugar biosynthetic genes are indicated by dark and light green arrows, respectively. Formyltransferases, methyltransferases, acetyltransferases and aminotransferases are encoded by genes represented by dark blue, purple, light blue and yellow arrows, respectively. Pink arrows indicates genes involved in fatty acid biosynthesis. Flanking genes are indicated by grey arrows, genes coding for hypothetical proteins or involved in functions with no relative known function in flagellin glycosylation by white arrows and black arrows indicate transposes and endonuclease genes. (TIF 3168 kb) [file 12864_2016_2735_MOESM3_ESM.tif]
